# Neutrophil CD64 index in cerebrospinal fluid as a marker of bacterial ventriculitis in children with external ventricular drainage

**DOI:** 10.1186/s12887-019-1497-4

**Published:** 2019-04-25

**Authors:** Mojca Groselj-Grenc, Metka Derganc, Andreja Natasa Kopitar, Maja Pavcnik

**Affiliations:** 1 Department of Paediatric Surgery and Intensive Care, University Medical Centre, Bohoriceva 20, 1525 Ljubljana, Slovenia; 2 Institute of Microbiology and Immunology, Faculty of Medicine, University of Ljubljana, Zaloska 4, 1000 Ljubljana, Slovenia; 3 Faculty of Medicine – Division of Pediatrics, University of Ljubljana, Vrazov trg 2, 1104 Ljubljana, Slovenia

**Keywords:** Bacterial ventriculitis, External ventricular drainage, Neutrophil CD64 index, Procalcitonin, C-reactive protein, Leukocyte count

## Abstract

**Background:**

Bacterial ventriculitis is a common complication in children with temporary external ventricular drains (EVD) and the diagnosis is challenging. The present study compared the diagnostic accuracy of novel cerebrospinal fluid (CSF) marker - CD64 expression on neutrophils measured as neutrophil CD64 index (CD64in) to routine laboratory CSF and blood markers for bacterial ventriculitis in children with EVD.

**Methods:**

We conducted a prospective, observational study, enrolling children with EVD. CD64in in CSF together with CSF markers (leukocyte count, percentage of neutrophils, glucose, and proteins) and blood markers (leukocyte and differential count, C-reactive protein (CRP), and procalcitonin (PCT)) were studied at the time of suspected bacterial ventriculitis. CD64in was measured by flow cytometry. Diagnostic accuracy determined by the area under the receiver–operating characteristic (ROC) curves (AUC) was defined for each marker.

**Results:**

Thirty-three episodes of clinically suspected ventriculitis in twenty-one children were observed during a 26-month period. Episodes were classified into those with microbiologically proven ventriculitis (13 episodes) and into those with microbiologically negative CSF (20 episodes). CD64in and leukocyte count were the only CSF markers that could differentiate between groups with diagnostic accuracy of 0.875 and 0.694, respectively. Among blood markers only CRP and band neutrophils differentiated between groups with diagnostic accuracy of 0.792 and 0.721, respectively.

**Conclusions:**

CD64in in CSF is a promising diagnostic marker of bacterial ventriculitis in children with EVD as it has higher diagnostic accuracy than routine blood and CSF markers for diagnosing bacterial ventriculitis at the time of clinical suspicion.

## Background

External ventricular drains (EVD), which divert cerebrospinal fluid (CSF) externally, are often used in children as temporary emergency tools to control raised intracranial pressure secondary to acute hydrocephalus caused by intracranial haemorrhage, neoplasm obstruction of the CSF circulation, or trauma [1]. Bacterial ventriculitis is a common complication of EVD, which increases morbidity and mortality, and prolongs hospitalization in these children [2–4]. Intraventricular or subarachnoid haemorrhage, cranial fracture with CSF leak, drain irrigation, craniotomy and duration of catheterization (more than 5 days) are all associated with increased risk of infection associated with EVD [1]. Most data on incidence of ventriculitis exists from studies of children with long-term ventricular shunts, in whom infection occurred in 5–15% [5–9]. Data on incidence of bacterial ventriculitis in children with temporary EVD are sparse and mostly available from mixed adult and children populations, in which bacterial ventriculitis affects up to 20% of patients with EVD [3, 10, 11].

Diagnosing bacterial ventriculitis in children with EVD is challenging due to frequent reoperations, blood contamination of CSF, presence of chemical ventriculitis and elevation of blood laboratory markers by concomitant systemic infection [4, 12]. Diagnosis of bacterial ventriculitis is based on clinical signs, laboratory markers of CSF and blood, and microbiological tests [13].

Clinical signs can be confounded by the primary neurological insult, critical care treatment (analgesia, sedation, and neuromuscular blockade), seizures, electrolyte disturbances and non-surgical infection [4]. Changes in routine cerebrospinal fluid parameters (leukocyte count, percentage of neutrophils, glucose, proteins) are often subtle, and difficult to interpret, since abnormalities can be related to placement of device, previous neurosurgery or infection [1, 14, 15].

Some novel markers of bacterial infection were already studied in CSF with promising results [16–21]. Routine blood markers of infection (leukocyte count, differential count, C-reactive protein (CRP) and procalcitonin (PCT)) may not be elevated early in the course of ventriculitis or are elevated because of drain placement, haemorrhage or concomitant systemic infection.

In an adult population with EVD early high serum PCT is the most reliable indicator of bacterial ventriculitis [1, 22, 23]. There are no reliable studies about blood markers in children with EVD to our knowledge. CSF cultures are still the most important test to establish the diagnosis of bacterial ventriculitis, but they take time to positive result and several days of incubation may be needed [1]. Novel diagnostic tools such as tests based on polymerase chain reaction may both increase the ability to identify a pathogen and decrease the time to specific diagnosis, but more studies are needed before routine use of these methods in children with EVD [1].

An additional CSF marker with high diagnostic accuracy for bacterial ventriculitis would be of great value. CD64 is a high-affinity and restricted isotype-specificity FcγRI receptor on neutrophils. Its expression is substantially up-regulated during infections, induced by inflammatory cytokines interferon-gamma and granulocyte colony-stimulating factor [24]. Until recently CD64 was studied mainly on blood neutrophils in different bacterial infections, but during bacterial ventriculitis the number of neutrophils in CSF increases and makes possible to measure CD64 also in CSF.

The present study compared the diagnostic accuracy of CD64 expression on neutrophils measured as neutrophil CD64 index (CD64in) to that of routine laboratory CSF (leukocyte count, percentage of neutrophils, glucose, proteins) and blood markers (leukocyte count, differential count, CRP and PCT) for bacterial ventriculitis in children with EVD.

## Methods

### Patients and setting

This prospective observational study was conducted at Department of Paediatric Surgery and Intensive Care of University Medical Centre Ljubljana, Slovenia during a 26-month period (December 2011–January 2014). Twenty-one consecutive patients with external ventricular drainage with thirty-three episodes of clinically suspected ventriculitis were eligible for enrolment. Clinical suspicion of ventriculitis was based on Centers for Disease Control and Prevention’s National Healthcare Safety Network (CDC/HSN) 2017 definition of meningitis or ventriculitis [13]. Inclusion criteria for the study were the presence of at least 1 of the clinical signs of ventriculitis: for children ≥1 year: fever > 38 °C, headache, meningeal sign(s) or cranial nerve sign(s) and for children ≤1 year: fever > 38 °C, hypothermia < 36 °C, apnoea, bradycardia, irritability, meningeal sign(s), or cranial nerve sign(s) combined with the initiation of a diagnostic workup for ventriculitis and the prescription of empirical antibiotic therapy. The diagnostic workup included routine laboratory tests (blood leukocyte and differential counts, CRP, PCT, CSF leukocyte and differential count, glucose, and proteins), and CSF culture with Gram stain, which were all obtained before antibiotic treatment was started. Patients were classified into two groups according to CSF culture: ventriculitis group and no-ventriculitis group. Ventriculitis group encompassed patients with positive CSF culture, and no-ventriculitis group patients with negative CSF culture in whom antibiotic therapy was discontinued after 2–3 days. The new episode of ventriculitis was defined, when at least one week had passed since previous episode and the child’s health improved during that week.

### Sample collection and measurements

CSF samples for flow cytometry (0.5 ml) were obtained at the time of suspected bacterial ventriculitis, together with samples for routine laboratory tests (CSF leukocyte count, glucose, proteins and blood leukocyte and differential count, CRP, PCT). CSF EDTA-anticoagulated samples were immediately transported to the flow cytometry laboratory or stored refrigerated (4 °C) during the nights and weekends up to 36 h, according to manufacturer’s reference and our previous study [25]. Expressions of CD64 and CD163 on neutrophils, monocytes and lymphocytes were measured by quantitative flow cytometry with a FACSCanto flow cytometer (Becton Dickinson, CD, USA) using the Leuko64™ assay (Trillium Diagnostics, LLC, Brewer, Maine, USA) [25, 26]. The sample preparation (50 μl of CSF) and flow cytometer setup were based on the manufacturer’s instructions and are described in details in our previous paper [26]. Index calculations were performed using Leuko64 QuantiCalc software (Trillium Diagnostics, LLC, Brewer, Maine, USA) and it was measured as a ratio of linearized mean fluorescence intensity of the cell population to the fluorescein isothiocyanate signal from the beads. Automated measurement of the lymphocyte CD64 index, which had to be less than 1.0, served as an internal negative control of the assay, and automated measurement of the monocyte CD64 index, which had to be more than 3.0, served as an internal positive control [26]. During working hours, the shortest time to obtain results is 2–3 h, and the price of 1 test is 38.5 EUR.

### Statistical analysis

Statistical analysis was performed using MedCalc for Windows, version 17.0.4 (MedCalc Software, Ostend, Belgium). Data were presented as the median with interquartile range (IQR). T-test for normally distributed data and Mann-Whitney test for not normally distributed data were used for comparing parametric variables between groups. Chi-square test was used for comparing nonparametric variables between groups. Receiver operating characteristic (ROC) curves for each studied marker were constructed to determine the optimal sensitivity, specificity, positive and negative predictive value, cut-off value and diagnostic accuracy from the area under the ROC curve (AUC). The cut-off values with the greatest sum of sensitivity and specificity were determined by the statistical program. Differences were considered to be statistically significant at a level of *p* < 0.05.

## Results

There were 13 episodes of microbiologically proven ventriculitis (ventriculitis group) and 20 episodes with microbiologically negative CSF (no-ventriculitis group). There were no statistically significant differences between groups in age, gender, and number of drainage days (*p* = 0.52, *p* = 0.37, and *p* = 0.75; respectively). The patients’ characteristics are summarized in Table 1. There were 9 Gram-positive pathogens isolated from CSF (*Staphylococcus epidermidis* 3, *Staphylococcus haemolyticus* 2, *Staphylococcus capitis* 1, *Streptococcus mitis* 1, *Streptococcus cristatus* 1, and *Enterococcus gallinarum* 1), and 4 Gram-negative pathogens (*Escherichia coli* 2, *Pseudomonas aeruginosa* 1, and *Serratia marcescens* 1).

**Table 1:** Characteristics of the study population

	Ventriculitis group	No-ventriculitis group
Number of episodes	13	20
Number of patients	12	16
*Median age (IQR)	9 months (5.5–112)	8.5 months (4–35)
Gender		
Male	7 (58%)	13 (81%)
Female	5 (42%)	3 (19%)
*Number of neonates (< 28 days)	2 (17%)	4 (25%)
*Number of infants (1–12 months)	5 (42%)	8 (50%)
*Median number of drainage days (IQR)	7 (3–18)	5.5 (1–18)
Type of hydrocephalus		
Congenital hydrocephalus	2 (17%)	3 (19%)
Post haemorrhagic hydrocephalus	8 (67%)	7 (44%)
Posttraumatic hydrocephalus	1 (8%)	2 (12%)
Post infectious hydrocephalus	0 (0%)	1 (6%)
Post tumour surgery hydrocephalus	1 (8%)	3 (19%)
Cause for EVD		
Hydro/haematocephalus de novo	7 (58%)	12 (75%)
Obstruction/infection of VPD	5 (42%)	3 (19%)
Liquor fistulae	0 (0%)	1 (6%)
*Positive Gram stain	8 (67%)	0 (0%)

*Values per episode; *IQR* – interquartile range, *EVD* – external ventricular drainage, *VPD* – ventriculo-peritoneal drainage, *CSF* – cerebrospinal fluid

**Fig. 1:**
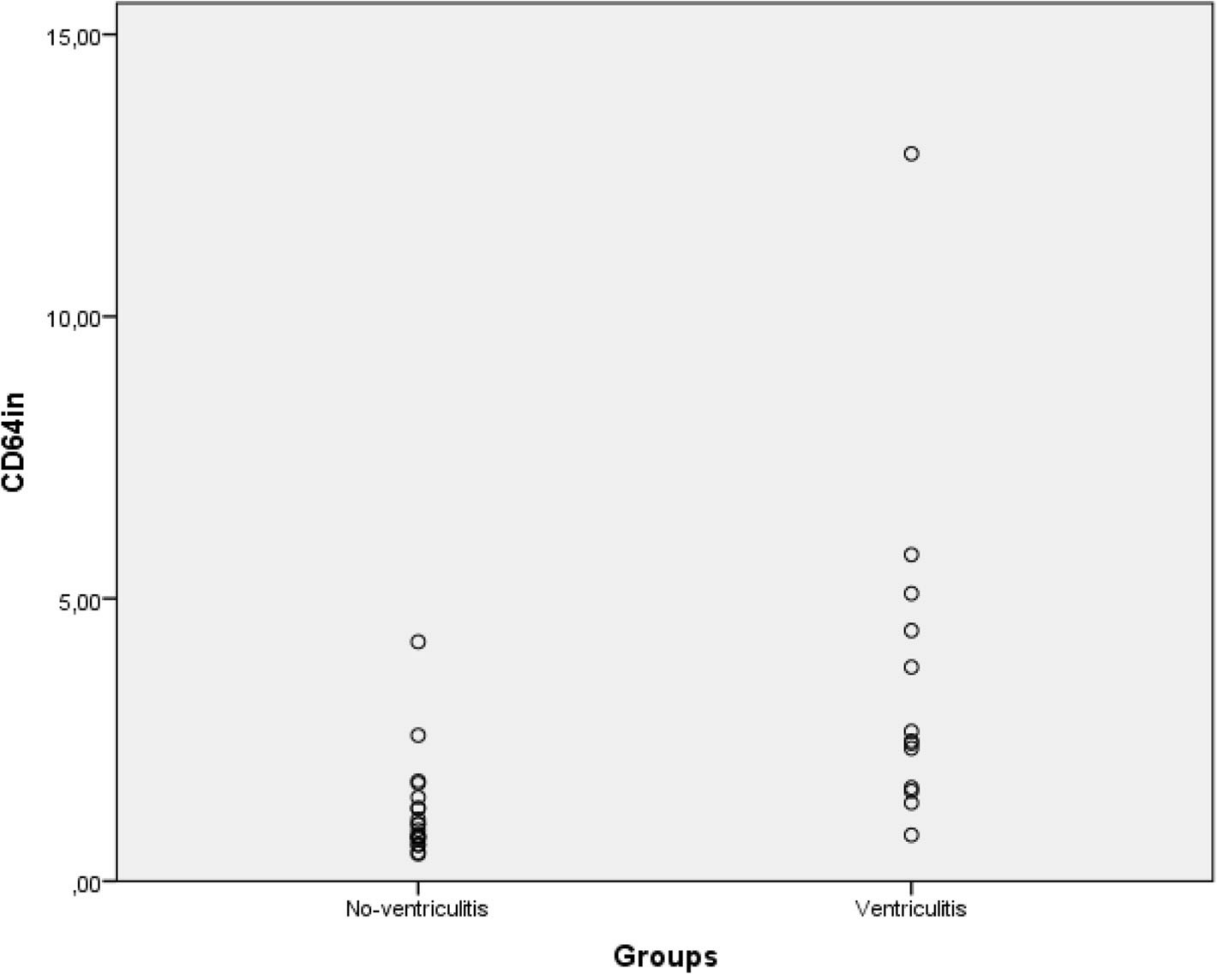
Individual values of CD64in in ventriculitis and no-ventriculitis group presented in a scatter diagram. CD64in – neutrophil CD64 index

**Fig. 2:**
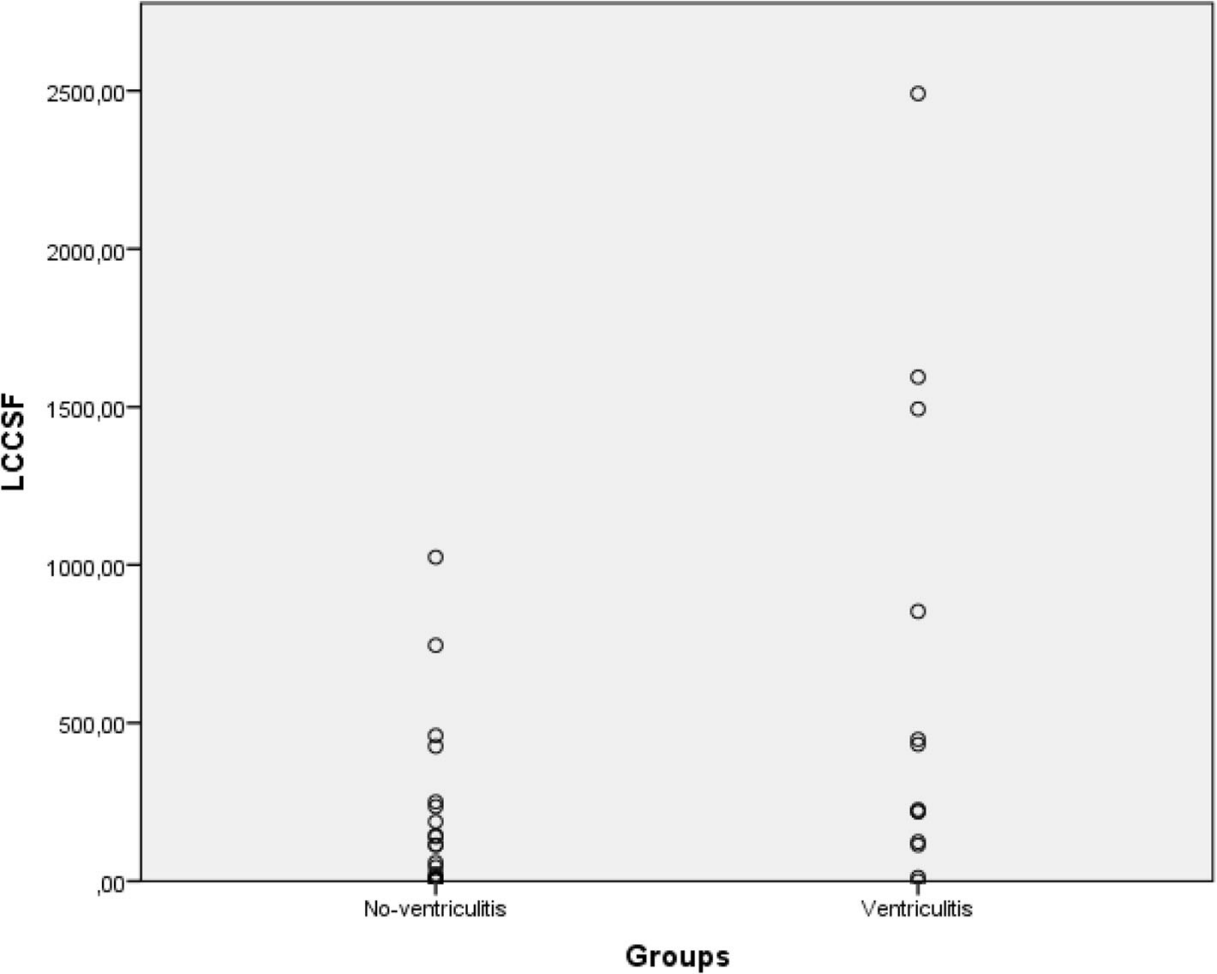
Individual values of LCCSF in ventriculitis and no-ventriculitis group presented in a scatter diagram. LCCSF – leukocyte count in cerebrospinal fluid

**Fig. 3:**
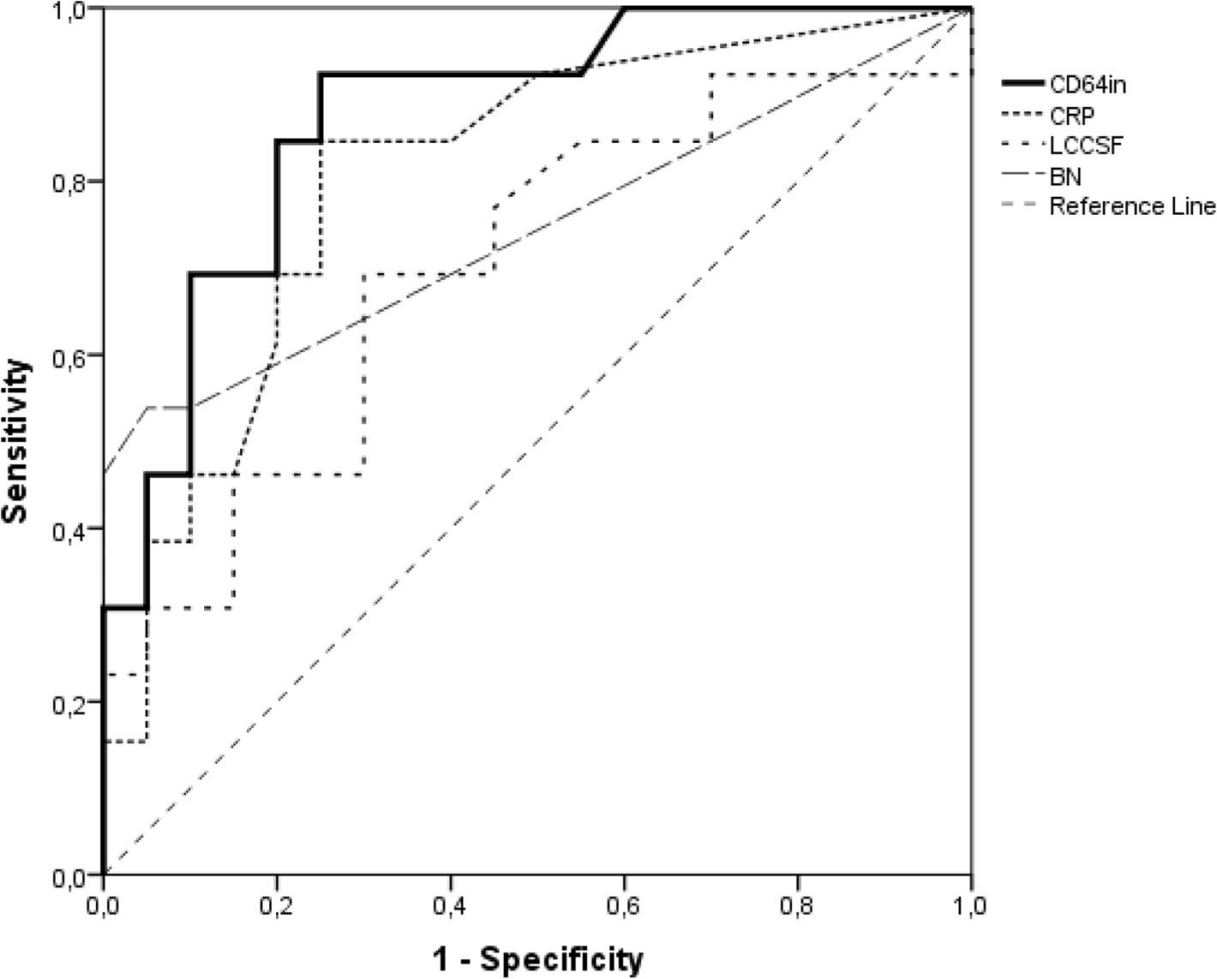
Receiver operating characteristic curves comparing CD64in in cerebrospinal fluid, blood CRP, LCCSF, and blood BN for prediction of bacterial ventriculitis in children. CD64in – neutrophil CD64 index; CRP – C-reactive protein; LCCSF – leukocyte count in cerebrospinal fluid; BN – band neutrophils

CD64in and leukocyte count were the only CSF markers that differentiated between groups; and among blood markers only CRP and band neutrophils differentiated between groups (Table 2). Figures 1 and 2 present individual values of CD64in and leukocyte count in CSF, respectively in ventriculitis and no-ventriculitis group, where modest overlapping between groups is observed for both markers. Table 3 shows the ROC curve analysis for blood and CSF laboratory markers. The highest diagnostic accuracy (0.875) was achieved by CD64in in CSF with high sensitivity (92%) and modest specificity (75%) at cut-off level 1.29, following by serum CRP (0.792) at cut-off level 5 mg/L. Figure 3 presents ROC curves illustrating the sensitivity and specificity for bacterial ventriculitis in children with EVD for markers with highest diagnostic accuracy for ventriculitis in CSF (CD64in, and leukocyte count in CSF) and blood (CRP, and band neutrophils).

**Table 2:** Median values with interquartile range of cerebrospinal fluid markers (CD64in, leukocyte count, percentage of neutrophils, glucose, proteins), and blood markers (leukocyte count, percentage of neutrophils and band neutrophils, CRP, and PCT) in ventriculitis and no-ventriculitis group

Laboratory markers	Ventriculitis group Median (IQR)	No-ventriculitis group Median (IQR)	*p*
CSF markers			
CD64in	2.48 (1.64–4.6)	0.95 (0.75–1.39)	< 0.01*
Leukocyte count (× 10^6^/L)	225 (0–2491)	113 (1–1024)	0.03*
Percentage of neutrophils (%)	66 (0–96)	50 (0–99)	0.27
Glucose (mmol/L)	1.6 (0.1–4.1)	2.3 (0.8–6.7)	0.12
Proteins (g/L)	1.51 (0.46–4.2)	0.79 (0.19–4.85)	0.14
Blood markers			
Leukocyte count (× 10^9^/L)	10.8 (5.1–26.8)	11.1 (2.7–18.3)	0.97
Percentage of neutrophils (%)	57 (22–86)	45 (15–81)	0.08
Percentage of band neutrophils (%)	2 (0–8)	0 (0–2)	0.05*
CRP (mg/L)	12 (0–330)	1.5 (0–162)	< 0.01*
PCT (μg/L)	0.25 (0.13–13.4)	0.17 (0.04–0.57)	0.14

*statistically significant differences, *p* < 0.05; *IQR* – interquartile range, *CSF* – cerebrospinal fluid, *CD64in* – neutrophil CD64 index, *CRP* – C-reactive protein, *PCT* – procalcitonin

**Table 3:** Diagnostic value of cerebrospinal fluid markers (CD64in, leukocyte count, percentage of neutrophils, glucose, and proteins, and blood markers (leukocyte count, percentage of neutrophils and band neutrophils, CRP, and PCT) for bacterial ventriculitis

	Optimum diagnostic cut-off level	AUC (95% CI)	Sensitivity (%)	Specificity (%)	PPV (%)	NPV (%)
Cerebrospinal fluid markers						
CD64in	1.29	0.875 (0.713–0.963)	92	75	71	94
Leukocyte count (×10^6^/L)	187	0.694 (0.507–0.844)	69	68	60	77
Percentage of N (%)	35	0.615 (0.428–0.781)	92	47	55	90
Glucose (mmol/L)	2.1	0.666 (0.478–0.822)	77	53	53	77
Proteins (g/L)	0.41	0.658 (0.470–0.815)	100	32	50	100
Blood markers						
Leukocyte count (×10^9^/L)	14.4	0.504 (0.325–0.682)	39	95	83	70
Percentage of N (%)	55	0.694 (0.496–0.850)	50	82	67	70
Percentage of BN (%)	2	0.721 (0.524–0.870)	50	100	100	74
CRP (mg/L)	5	0.792 (0.616–0.913)	85	75	69	88
PCT (μg/L)	0.17	0.716 (0.507–0.873)	85	54	65	78

*AUC* – area under the receiver operating curve, *CI* – confidence interval, *PPV* – positive predictive value, *NPV* – negative predictive value, *CD64in* – neutrophil CD64 index, *N* – neutrophils, *BN* – band neutrophils, *CRP* – C-reactive protein, *PCT* – procalcitonin

## Discussion

Diagnostic approach to children with EVD and clinical suspicion of bacterial ventriculitis is based on microbiological techniques and laboratory markers of infection, which can be studied in blood and CSF. In the presented study, CD64in - a novel CSF marker of bacterial ventriculitis in children with EVD, was compared to routine blood and CSF markers. We showed that CD64in is better marker for bacterial ventriculitis than routine markers.

CD64 on blood neutrophils has already been shown to be highly accurate for diagnosis of bacterial infection and sepsis in neonates, children, adults and in surgical patients [24, 25, 27, 28]. To our knowledge there is only one report of blood neutrophil CD64 in bacterial meningitis in the literature; it showed 100% sensitivity and 65% specificity for bacterial meningitis in group of 132 children with suspected meningitis [29]. There is no data on CD64 on CSF neutrophils during bacterial infection of central nervous system. In our present study CD64in was significantly higher in children with bacterial ventriculitis compared to those without ventriculitis. Only leukocyte count in CSF, and CRP and percentage of band neutrophils in blood could also differentiated between these two groups. There are few data about routine CSF and blood tests in bacterial ventriculitis in children. In the study of Schumann and co-authors CRP was studied in serum and CSF of children with suspected internal ventricular shunt infection. Serum CRP was found superior to CSF CRP and all other routine markers [30]. A large multicentre study comparison of CSF in neonates with and without ventricular shunts, who had undergone lumbar puncture, revealed that neonates with negative CSF and ventricular shunts had significantly higher red blood cell count, protein level and lower glucose level in comparison to neonates with negative CSF and no ventricular shunts [14]. When analysing CSF positive cases, the authors concluded, that utility of routine CSF markers in neonates with ventricular shunts is limited [14].

Diagnostic accuracy of CD64in in our study was higher than diagnostic accuracy of any routine CSF or blood markers. CRP had the second highest diagnostic accuracy in our study; it was moderately lower than the one found by Schumann et al. for serum CRP (0.916) in children with internal ventricular shunts [30]. In adults with EVD, serum PCT is superior to CRP for diagnosing bacterial ventriculitis [22]. In our study diagnostic accuracy of PCT was lower than that of CRP, probably because of different study design and different population. We believe that in our study laboratory markers have been obtained much sooner, immediately after clinical suspicion of bacterial ventriculitis, since median levels of blood laboratory markers have been quite low. Cut-off level for CD64in in CSF for bacterial ventriculitis in our present study was lower than cut-off level for sepsis in sera of neonates and children in our previous study, while diagnostic accuracy of CD64in for ventriculitis was between that of neonates and children for sepsis at the time of sepsis suspicion [25].

Several novel CSF markers of bacterial ventriculitis in children and adults have already been studied. A cytokine interleukin-6 (IL-6) was found to be a reliable marker for prediction of bacterial infection in adult neurosurgical patients with EVD after subarachnoid haemorrhage, when performed on daily basis [19, 20]. In other studies IL-6 failed to diagnose bacterial ventriculitis [15, 21]. Soluble triggering receptor expressed on myeloid cells-1 (TREM-1) was found to be similarly useful for diagnosing bacterial ventriculitis in adults with EVD compared to classical CSF markers [17]. In another study decreased expression of neutrophil CD62L was found to increase specificity and sensitivity for bacterial ventriculitis in adults with blood containing CSF and EVD [16]. Lopez-Cortes et al. studied a myriad of CSF markers and found interleukin-1beta (IL-1beta) as a best marker for CSF infection in mixed neurosurgical population of adults and children [21]. In our previous study in children with EVD, presepsin (sCD14-ST) showed highest diagnostic accuracy (0.877), when compared to routine CSF and blood markers and its diagnostic accuracy was very similar to diagnostic accuracy of CD64in in our present study [18].

Gram-positive bacteria, mainly coagulase-negative staphylococci, were predominant causative pathogens in our children with ventriculitis. Coagulase-negative staphylococci can be considered as contaminants in adults but not in neonates and infants, in whom they can cause serious infection [31, 32]. The majority of our patients were neonates and infants. Similar to our results, some other authors found coagulase-negative staphylococci to be main pathogens in adult patients with ventriculitis (56%) [33, 34]. It is believed that Gram-positive cocci that are part of skin flora are the most common pathogens causing nosocomial ventriculitis and meningitis in neuro-critical care patients [35].

Several limitations of this study merit consideration. Firstly, diagnosis of bacterial ventriculitis was based on results of microbiological cultures of CSF. Although only children with clinical signs of infection were included, it is still possible that some cases of colonisation were recognized as infection. Gram stain is usually done at the time of suspicion of bacterial ventriculitis at our institution, since the result is quickly obtained, but negative result does not exclude bacterial infection [36]. Gram stain has high specificity, but low sensitivity and was found positive in only 54% of children with positive CSF culture [30]. In our study Gram stain was positive in only 62% of children with culture proven ventriculitis. Novel microbiological methods based on polymerase chain reaction (PCR) can show higher diagnostic accuracy than cultures with shorter time to results, but they still lack the evaluation in larger studies and are not yet performed routinely [1, 37]. These methods can be especially problematic in children with EVD, who usually have many consecutive episodes of infection during their stay on EVD with prolonged antibiotic treatment [1]. PCR methods still need to be improved to differentiate between infection, ventricular drain colonization, sample contamination and nonviable organisms due to antibiotic treatment or previous infection [38]. Secondly, it is possible that some CSF infection with slow growing bacteria was missed after routine 2–3 days antibiotic treatment in our patients and discovered as ventriculitis in next episode. Thirdly, group of children, who need EVD insertion is usually heterogeneous and many factors should be considered when interpreting the results of CSF analysis (time of previous surgery, time of EVD insertion, age and gestational age in neonates, blood contamination of CSF, etc), which could all influence the results and make the interpretation difficult. Besides, blood glucose levels were missing in our children, therefore comparison between CSF and blood glucose levels was not possible. Finally, we studied a relatively small number of patients from a single institution. The number of children with EVD and time spent with EVD was recently reduced at our institution since introduction of new surgical methods for bridging the time for CSF clearing before implantation of internal ventricular shunting, such as ventriculo-subgaleal shunts. A larger study, perhaps multicentred, which would include children with internal ventricular shunts, is warranted in the future.

## Conclusions

Our study showed that CD64in has the highest diagnostic accuracy among all routine blood and CSF markers for diagnosing bacterial ventriculitis in children with EVD. Since clinical diagnosis is very difficult in this specific population, a novel CSF marker with high negative predictive value, such as CD64, would be useful when added to routine markers for differentiating children who require antibiotic treatment and taking microbiological culture, from those who do not. Furthermore, since many novel CSF markers have recently been studied with promising results, a combination of these markers, including CD64, would be of great value in this population. With simplification and extension of availability of flow cytometry, CD64in measurement might be routinely done in these children, but because of very challenging diagnosis of ventriculitis in this population, it should be viewed with caution.

## Abbreviations

AUC: Area under the curve
BN: Band neutrophils
CD64in: Neutrophil CD64 index
CDC/HSN: Centers for Disease Control and Prevention’s National Healthcare Safety Network
CI: Confidence interval
CRP: C-reactive protein
CSF: Cerebrospinal fluid
EVD: External ventricular drains
IL-1beta: Interleukin-1beta
IL-6: Interleukin-6
IQR: Interquartile range
LCCSF: Leukocyte count in cerebrospinal fluid
N: Neutrophils
NPV: Negative predictive value
PCT: Procalcitonin
PPV: Positive predictive value
ROC: Receiver–operating characteristic
sCD14-ST: presepsin
TREM-1: Soluble triggering receptor expressed on myeloid cells-1
VPD: Ventriculo-peritoneal drainage

## References

[1] Tunkel AR, Hasbun R, Bhimraj A, et al. Infectious Diseases Society of America's clinical practice guidelines for healthcare-associated Ventriculitis and meningitis. Clin Infect Dis. 2017; Epub 2017 Feb 14. 10.1093/cid/ciw861 PMC5848239 28203777

[2] Pfausler B, Spiss H, Beer R (2003). Treatment of staphylococcal ventriculitis associated with external cerebrospinal fluid drains: a prospective randomized trial of intravenous compared with intraventricular vancomycin therapy. J Neurosurg.

[3] Phan K, Schultz K, Huang C (2016). External ventricular drain infections at the Canberra hospital: a retrospective study. J Clin Neurosci.

[4] Ramanan M, Lipman J, Shorr A, Shankar A (2015). A meta-analysis of ventriculostomy-associated cerebrospinal fluid infections. BMC Infect Dis.

[5] Attenello FJ, Garces-Ambrossi GL, Zaidi HA, Sciubba DM, Jallo GI (2010). Hospital costs associated with shunt infections in patients receiving antibiotic-impregnated shunt catheters versus standard shunt catheters. Neurosurgery..

[6] Arnell K, Cesarini K, Lagerqvist-Widh A, Wester T, Sjölin J (2008). Cerebrospinal fluid shunt infections in children over a 13-year period: anaerobic cultures and comparison of clinical signs of infection with Propionibacterium acnes and with other bacteria. J Neurosurg Pediatr..

[7] Wang KW, Chang WN, Shih TY (2004). Infection of cerebrospinal fluid shunts: causative pathogens, clinical features, and outcomes. Jpn J Infect Dis.

[8] Sciubba DM, Lin LM, Woodworth GF, McGirt MJ, Carson B, Jallo GI (2007). Factors contributing to the medical costs of cerebrospinal fluid shunt infection treatment in pediatric patients with standard shunt components compared with those in patients with antibiotic impregnated components. Neurosurg Focus.

[9] Rowensztein H, Manfrin L, Paglia M, Cong TL, Ruvinsky S, Scrigni A (2015). Characteristics of cerebrospinal fluid (CSF) among children with ventriculoperitoneal shunt infections. Arch Argent Pediatr.

[10] Camacho EF, Boszczowski I, Basso M (2011). Infection rate and risk factors associated with infections related to external ventricular drain. Infection..

[11] Leverstein-van Hall MA, Hopmans TE, van der Sprenkel JW (2010). A bundle approach to reduce the incidence of external ventricular and lumbar drain-related infections. J Neurosurg.

[12] Miller C, Guillaume D (2015). Incidence of hemorrhage in the pediatric population with placement and removal of external ventricular drains. J Neurosurg Pediatr.

[13] CDC/NHSN Surveillance Definitions for Specific Types of Infections January 2017 [Internet]. Atlanta (GA): Centers for Disease Control and Prevention. [cited 2017 Aug 27]. Available from: https://www.cdc.gov/nhsn/pdfs/pscmanual/17pscnosinfdef_current.pdf

[14] Lenfestey RW, Smith PB, Moody MA (2007). Predictive value of cerebrospinal fluid parameters in neonates with intraventricular drainage devices. J Neurosurg.

[15] Schade RP, Schinkel J, Roelandse FW (2006). Lack of value of routine analysis of cerebrospinal fluid for prediction and diagnosis of external drainage-related bacterial meningitis. J Neurosurg.

[16] Boer K, Vogelsang H, Deufel T, Pfister W, Kiehntopf M (2010). CD62L on neutrophil granulocytes, a useful, complementary marker for the prediction of ventriculitis in blood-containing CSF. Clin Biochem.

[17] Gordon M, Ramirez P, Soriano A (2014). Diagnosing external ventricular drain-related ventriculitis by means of local inflammatory response: soluble triggering receptor expressed on myeloid cells-1. Crit Care.

[18] Stubljar D, Kopitar AN, Groselj-Grenc M, Suhadolc K, Fabjan T, Skvarc M (2015). Diagnostic accuracy of presepsin (sCD14-ST) for prediction of bacterial infection in cerebrospinal fluid samples from children with suspected bacterial meningitis or ventriculitis. J Clin Microbiol.

[19] Schoch B, Regel JP, Nierhaus A (2008). Predictive value of intrathecal interleukin-6 for ventriculostomy-related infection. Zentralbl Neurochir.

[20] Lenski M, Huge V, Briegel J, Tonn JC, Schichor C, Thon N (2017). Interleukin 6 in the cerebrospinal fluid as a biomarker for onset of vasospasm and ventriculitis after severe subarachnoid hemorrhage. World Neurosurg.

[21] López-Cortés LF, Marquez-Arbizu R, Jimenez-Jimenez LM (2000). Cerebrospinal fluid tumor necrosis factor-alpha, interleukin-1beta, interleukin-6, and interleukin-8 as diagnostic markers of cerebrospinal fluid infection in neurosurgical patients. Crit Care Med.

[22] Berger C, Schwarz S, Schaebitz WR, Aschoff A, Schwab S (2002). Serum procalcitonin in cerebral ventriculitis. Crit Care Med.

[23] Omar AS, ElShawarby A, Singh R (2015). Early monitoring of ventriculostomy-related infections with procalcitonin in patients with ventricular drains. J Clin Monit Comput.

[24] Nuutila J (2010). The novel applications of the quantitative analysis of neutrophil cell surface FcgammaRI (CD64) to the diagnosis of infectious and inflammatory diseases. Curr Opin Infect Dis.

[25] Groselj-Grenc M, Ihan A, Pavcnik-Arnol M, Kopitar AN, Gmeiner-Stopar T, Derganc M (2009). Neutrophil and monocyte CD64 indexes, lipopolysaccharide-binding protein, procalcitonin and C-reactive protein in sepsis of critically ill neonates and children. Intensive Care Med.

[26] Groselj-Grenc M, Ihan A, Derganc M. Neutrophil and monocyte CD64 and CD163 expression in critically ill neonates and children with sepsis: comparison of fluorescence intensities and calculated indexes. Mediat Inflamm. 2008;202646. 10.1155/2008/202646 PMC2442385 18604302

[27] Liu Y, Hou JH, Li Q, Chen KJ, Wang SN, Wang JM (2016). Biomarkers for diagnosis of sepsis in patients with systemic inflammatory response syndrome: a systematic review and meta-analysis. Springerplus..

[28] Streimish I, Bizzarro M, Northrup V (2012). Neutrophil CD64 as a diagnostic marker in neonatal sepsis. Pediatr Infect Dis J.

[29] Mohamed HB, Alif HA, Awadalla AA, Azza ZL (2012). Detection and significance of blood neutrophil CD64 expression as a diagnostic marker in bacterial meningitis in children. Egypt J Immunol.

[30] Schuhmann MU, Ostrowski KR, Draper EJ (2005). The value of C-reactive protein in the management of shunt infections. J Neurosurg.

[31] Wiegand J, Hickson L, Merz TM (2016). Indicators of external ventricular drainage-related infections–a retrospective observational study. Acta Neurochir.

[32] Marchant EA, Boyce GK, Sadarangani M, Lavoie PM. Neonatal sepsis due to coagulase-negative staphylococci. Clin Dev Immunol. 2013;586076. 10.1155/2013/586076 PMC3674645 23762094

[33] Scheithauer S, Bürgel U, Ryang YM (2009). Prospective surveillance of drain associated meningitis/ventriculitis in a neurosurgery and neurological intensive care unit. J Neurol Neurosurg Psychiatry.

[34] Hoogmoed J, van de Beek D, Coert BA, Horn J, Vandertop WP, Verbaan D (2017). Clinical and laboratory characteristics for the diagnosis of bacterial ventriculitis after aneurysmal subarachnoid hemorrhage. Neurocrit Care.

[35] Beer R, Lackner P, Pfausler B, Schmutzhard E (2008). Nosocomial ventriculitis and meningitis in neurocritical care patients. J Neurol.

[36] He T, Kaplan S, Kamboj M, Tang YW (2016). Laboratory diagnosis of central nervous system infection. Curr Infect Dis Rep.

[37] Rath PM, Schoch B, Adamzik M, Steinmann E, Buer J, Steinmann J (2014). Value of multiplex PCR using cerebrospinal fluid for the diagnosis of ventriculostomy-related meningitis in neurosurgery patients. Infection..

[38] Gordon CL, Tokarz R, Briese T (2015). Evaluation of a multiplex polymerase chain reaction for early diagnosis of ventriculostomy-related infections. J Neurosurg.

